# “Imagine a pregnancy”: perspectives of Latine emerging adults from an agricultural community in California

**DOI:** 10.3389/frph.2025.1720373

**Published:** 2026-01-09

**Authors:** Amanda E. Bryson, Paula S. Nordstrom Miranda, Melissa S. Zerofsky, Alondra Jamie-Aguilar, Mary Kate Shapley-Quinn, Alexandra Minnis, Marissa Raymond-Flesch

**Affiliations:** 1 Department of Pediatrics, University of California, San Francisco, CA, United States; 2 School of Medicine, University of California San Francisco, San Francisco, CA, United States; 3 School of Public Health, University of California Berkeley, Berkeley, CA, United States; 4 Women’s Global Health Imperative, RTI International, Oakland, CA, United States; 5 Philip R. Lee Institute for Health Policy Studies, University of California, San Francisco, CA, United States

**Keywords:** decision making, emerging adult, Hispanic or Latino, pregnancy, young adults

## Abstract

**Introduction:**

Pregnancy perceptions and pregnancy acceptability have been identified as alternative multidimensional constructs to elucidate and integrate people's lived experiences, needs, and goals related to reproduction, pregnancy, and parenting. This study examines the perspectives of Latine emerging adults on a hypothetical pregnancy and what socioecological factors would influence pregnancy-related decisions.

**Methods:**

In a mixed-methods prospective cohort study of emerging adults from an agricultural community in California followed since eighth grade, interviews were conducted with a subset of participants (5/2023–1/2024). In the interviews, participants were asked to reflect on a hypothetical pregnancy. Qualitative data analysis was performed using directed content and inductive analyses of the interview transcripts. Descriptive statistics were used to complement the qualitative findings and describe the participants' demographics, characteristics, and pregnancy desire.

**Results:**

Forty-one participants (ages 19–21 years; *N* = 20 female, *N* = 17 male, *N* = 4 non-binary; 12% first generation, 71% second generation, and 17% third generation immigrants) were interviewed. Most participants (*N* = 30) reported that they really or mostly did not want to get pregnant or get a partner pregnant now or in the next few months. When asked about a hypothetical pregnancy, most participants discussed continuing the pregnancy and parenting or having an abortion as their preferred pregnancy option. When discussing hypothetical pregnancies and related decisions, participants discussed influences across socioecological levels, including individual (reactions, maturity, readiness, finances, life trajectory), interpersonal (partners, friends, parents, other family members), and community and systems (norms, culture, laws, politics, religion, healthcare access).

**Conclusions:**

These findings deepen our understanding of the influences on Latine emerging adults' perspectives of pregnancy and related decisions, which can inform the development of interventions at different socioecological levels to help individuals realize their reproductive goals.

## 1 Introduction

Pregnancies are often characterized as “intended” or “unintended” in research and medicine. However, many pregnancies are perceived as somewhere in between. Moreover, the concept of pregnancy intention does not necessarily reflect a person's complex attitudes towards childbearing (1–5). Pregnancy intentions rely on a pregnancy planning paradigm which may not be salient or attainable given cultural norms and structural inequities (1, 3, 6, 7). Research has demonstrated that, while high preference to avoid pregnancy correlates with contraceptive use and consistency of use (8, 9), many people with a low preference to avoid pregnancy also use contraception (8). This suggests that desire to prevent pregnancy does not predict desire or need to use contraceptives (4, 8). While pregnancy intendedness or desire to prevent pregnancy can be important factors informing sexual behaviors and reproductive health decisions, these findings suggest that other factors contribute to contraceptive decision making. Given these limitations of using pregnancy intentions as a primary approach to informing contraceptive needs, experts have advocated for the investigation of more holistic and person-centered approaches to help people achieve their reproductive goals (1, 4, 10).

Studies have investigated the relationship between pregnancy intentions and feelings about a pregnancy among Latine individuals living in the United States, with results suggesting that pregnancy intentions and feelings about pregnancy are distinct concepts. Studies utilizing large nationally representative data reveal that Latine men and women are more likely report positive feelings about pregnancy, regardless of intention, than those who identify as white or Black (11–13). These sentiments are reflected in qualitative studies interviewing adult women, most of whom identified as Latina, where more than two-thirds of participants had incongruent pregnancy intentions and feelings about the prospect of a pregnancy (14).

Several studies investigating the relationship of pregnancy intentions, pregnancy acceptability, and perception of childbearing among Latine adolescents and young adults (AYAs) have demonstrated the complexity of these constructs in this population. Similar to research of Latine adults, Latine adolescents are more likely to report that they would be “very pleased” if they or their partner became pregnant than non-Latine white and non-Latine Black adolescents (15) and endorse discordant pregnancy intentions and feelings related to pregnancy (e.g., acceptability, happiness) (10, 16). Additionally, a longitudinal study of urban Latine adolescents found that pregnancy intentions fluctuated over time and that intentions did not predict pregnancies among this sample, except at the highest levels of intention (16). These findings suggest that factors, other than pregnancy intentions, are influencing reproductive outcomes for Latine adolescents, including feelings and beliefs about childbearing and cultural norms (16). One quantitative study found that perceived benefits of childbearing influenced AYAs' incidence of pregnancy over one year (2). Perceived benefits of childbearing may be particularly influential among Latine AYAs as they perceived more benefits to childbearing than their white counterparts (2). The existing literature highlights a need to better understand Latine AYAs' perceptions and preferences about pregnancy and childbearing, as well as what influences pregnancy-related decisions to better elucidate what is needed to achieve their reproductive goals.

More recently, pregnancy perceptions and pregnancy acceptability have been identified as alternative multidimensional constructs to elucidate and integrate people's lived experiences, needs, and goals related to reproduction, pregnancy, and parenting (1, 10). A published conceptual model describes the relationship between one's perception of pregnancy (e.g., desires, emotions, and intentions), socioecological factors (e.g., internal factors, partner dynamics, social/economic/cultural environment, and the healthcare system), and sexual health behaviors (e.g., using contraception, preparing for possible pregnancy, seeking information) (1). In line with this model, this community-engaged qualitative research study, rooted in the socioecological model (which accounts for the multiple environmental layers that influence health outcomes) (17), explores the pregnancy perspectives of Latine emerging adults (ages 19 to 21 years) from a rural agricultural community in California. Through in-depth interviews we sought to examine participants' perspectives on a hypothetical pregnancy now and what socioecological factors would influence their decisions related to pregnancy.

## 2 Materials and methods

We report on findings from the in-depth interviews of the *A Crecer* study, a community-engaged, mixed methods, longitudinal cohort study of emerging adults followed over eight years since enrollment in eighth grade from Salinas Valley in California's Central Coast. The setting and study design of the initial waves of data collection (waves one to five) in *A Crecer* have been previously described (18, 19). At initial enrollment of the study (wave one), eligible participants were aged 12–15 years old, able to complete the study procedures in English or Spanish, intended to live in Salinas for the next year, and were willing to provide contact information for a parent. At wave one, 599 participants were enrolled, representing about one third of students in the Salinas Union High School District from which the study recruited participants. Of the 599 participants who were enrolled in the original cohort (waves one to five), 389 (65%) participants re-enrolled at wave six approximately six years after initial enrollment. The overarching goal of waves six to eight in the *A Crecer* study is to identify social and structural influences on sexual health outcomes among rural Latine youth as they transition from adolescence to emerging adulthood (ages 18–24). Annual study visits include a quantitative questionnaire and biological testing for pregnancy and two treatable sexually transmitted infections. In wave six of *A Crecer,* 389 participants re-enrolled and completed the study visit. A subset of participants (*N* = 41) completed an in-depth interview between wave six and seven visits. We aimed to recruit a sample size of approximately 40 participants to complete in-depth interviews based achieving thematic saturation with this sample size in the study's two prior waves of qualitative analyses. The RTI International Institutional Review Board (IRB) approved all study activities and methods.

### 2.1 Study design and population

After completing the *A Crecer* wave six study questionnaire, the research team invited a subset of participants for an in-depth interview, which occurred on average seven and a half months after completing the baseline questionnaire. The research team selected participants who represented a variety of recent relationship experiences in the last year with target numbers established to ensure participation among female, male and nonbinary-identified individuals. Relationship experiences included multiple partners, one partner (casual or serious), or no partner in the last year. Additionally, participants were selected to ensure a variety of sexual orientations (at least 3) were reflected across the different relationship experiences. Within these sampling criteria, the field team had autonomy to select which eligible participants to invite to complete an in-depth interview. The interview guide was designed to focus on sexual health decision making and communication in young adulthood. It included the question, “How would you feel or react if you found out you [your partner] were pregnant today?” Interviewers probed what pregnancy options the participant would consider, what factors would influence decisions related to pregnancy, and who they would turn to for support in this scenario. The analysis presented in this manuscript specifically focuses on the themes related to this question.

The research team conducted interviews in the participants' preferred language (English, *N* = 39; Spanish, *N* = 2) from May 27, 2023 to January 12, 2024 in a private room at the study office in downtown Salinas, California or via Zoom (In person, *N* = 39; Zoom, *N* = 2). All participants completed a consent process with a study interviewer prior to participation in the interview, providing signed consent. All interviewers were bilingual, bicultural and from California's Central Coast. The interviews were approximately 50 min on average (ranging 27–98 min). Participants received $40 compensation for completing the interview.

### 2.2 Data analysis

English interviews (*N* = 39) were transcribed verbatim via a professional transcription service, and Spanish interviews (*N* = 2) were transcribed via a one-step process where a certified bilingual transcriptionist listened to the audio recording in Spanish and translated in real-time to produce an English language transcript. The study's bilingual and bicultural research assistants performed quality checks on all interview transcripts against the original recordings, paying particular attention to ensuring linguistic equivalence for the translated transcripts. Research assistants incorporated brief descriptions of any idiomatic language that would be relevant for analysis in English or Spanish in the transcript prior to analysis. Transcribed transcripts were stored in Dedoose, a qualitative research software. Four members of the analysis team (AB, PNM, MZ, MRF) reviewed nine transcripts and created memos to identify initial themes, which were further developed and refined into codes using directed content and inductive analyses, informed by the socioecological model. All members of the analysis team applied the initial codebook to three transcripts during which time the codebook was discussed and refined. All transcripts were dual coded in Dedoose. Coding discrepancies and questions were discussed and resolved in weekly meetings. During weekly meetings, the analysis team reflected on our identities and biases as we engaged in coding and analysis. AB is a white-identifying cisgender woman who became involved with the *A Crecer Study* as of Fall 2023. MRF is second-generation Mexican American cisgender woman who has been involved in *A Crecer* since 2014. AB and MRF are physician scientists and practicing adolescent medicine physicians who provide reproductive healthcare including medication abortions. PNM is a second-generation, mixed-race, Peruvian American cisgender woman from rural Northern California who became involved with *A Crecer* in Fall 2023. PNM is a medical student who supports an individual's right to reproductive freedom, including the right to access abortion. MZ is a white-identifying cisgender woman who is a clinical research coordinator in Pediatrics and has been involved in *A Crecer* since 2020. The analysis team explored thematic areas through discussions in research meetings, creation of memos, and sub coding the data. We presented findings to the *A Crecer* Young Adult Advisory Board as a validity check and to further shape our interpretation of the data.

Demographic and quantitative data presented in this analysis were drawn from the *A Crecer* wave six and seven questionnaires to describe the sample. To further contextualize the participants' discussion of a hypothetical pregnancy, we also report a quantitative measure of pregnancy intentions by using data from a question asked on the *A Crecer* wave six questionnaire: “Now or in the next few months, how much do you want [a partner] to get pregnant?”. Responses were on a five-point Likert scale. We analyzed the data in Stata (StataCorp, LLC) using frequencies and percentages for categorical variables.

## 3 Results

### 3.1 Demographics

Forty-one participants (51% female, 44% male, and 5% nonbinary), almost all of whom identified as Latine (93%), completed in-depth interviews (Table 1). Most participants were in a relationship with one or more partners in the last 12 months (78%) and had vaginal sexual intercourse in the past 12 months (74%).

**Table 1:** Participants' demographics and characteristics, A Crecer study emerging adulthood qualitative sample, 2024 (*N* = 41).

Variable	*N*	%
Age (years)		
19	19	46
20	15	37
21	7	17
Gender identity		
Female	20	51
Nonbinary	4	5
Male	17	44
Identifies as Latine		
Yes	38	93
No	3	7
Mexican, Mexican American, Chicano origin		
Yes	37	90
No	4	10
Generation of US immigrant		
1st generation	5	12
2nd generation	29	71
3rd generation	7	17
Highest grade or level of school completion (*N* = 40)		
11th grade	1	3
12th grade, diploma	34	85
12th grade, no diploma	0	0
Some college	3	8
Associate degree	2	5
Currently in school		
Yes	21	51
No	20	49
Marital status		
Married	0	0
Living with a partner as an unmarried couple	5	12
None of these	36	88
Current work status		
Full-time	13	32
Part-time	16	39
Not working	12	29
Received cash assistance from a government-sponsored assistance program^a^		
Yes	14	34
No	26	63
Don't know	1	2
Received other types of help/assistance from the government^b^		
Yes	18	44
No	22	54
Don't know	1	2
Covered by health insurance (*N* = 37)		
Yes	33	89
No	4	11
Importance of religion		
Very important	6	15
Somewhat important	21	51
Not very important	5	12
Not important at all	9	22
Living with		
Mother	33	81
Father	23	56
Sibling(s)	29	71
Partner	5	12
Own children	3	7
Other children	5	12
Friend(s) or other adult roommate(s)	5	12
Someone else	7	17
Lives alone	1	2
Sexual orientation^c^		
Gay	1	2
Lesbian	0	0
Straight	30	73
Bisexual	4	10
Pansexual	2	5
Sapphic	1	2
Bi curious	1	2
Androgynous	1	2
Don't know	1	2
Relationship in the last 12 months		
No partner	9	22
One partner	17	41
More than one partner	15	37
Ever pregnant (self or partner)		
Yes^d^	4	10
No	37	90

Participants who skipped or didn't answer questions were excluded from the counts.

a Government-sponsored assistance programs included welfare, food stamps, and temporary assistance for needy families.

b Other help or assistance included Women, Infant, and Children program, public health insurance (Medi-Cal), or Supplemental Nutrition Assistance Program.

c Bi curious, Pansexual, Sapphic, and Androgynous were self-described answers.

d Three female participants had previous pregnancies, which resulted in two live births and one miscarriage. One male participant had a partner who became pregnant, which resulted in one live birth.

### 3.2 Quantitative data: desire for pregnancy

Participants had various levels of desire for a pregnancy now or in the next few months (Table 2). Most participants (*N* = 30) reported that they really or mostly did not want to get pregnant or get a partner pregnant now or in the next few months.

**Table 2:** Participants' responses to “now or in the next few months, how much do you want [a partner] to get pregnant?”, A Crecer study emerging adulthood qualitative sample, 2024.

Desire for pregnancy	*N*	%
Desire for pregnancy (self)		
Really DO NOT want to get pregnant	16	66.7
Mostly DO NOT want to get pregnant	1	4.2
Sometimes WANT to and sometimes DO NOT want to get pregnant	4	16.7
Mostly WANT to get pregnant	1	4.2
WANT to get pregnant	1	4.2
Don't know	1	4.2
Total	24	100
Desire for pregnancy (partner)		
Really DO NOT want to get a partner pregnant	11	68.8
Mostly DO NOT want to get a partner pregnant	2	12.5
Sometimes WANT to and sometimes DO NOT want to get a partner pregnant	2	12.5
Mostly WANT to get a partner pregnant	1	6.3
Really WANT to get a partner pregnant	0	0
Don't know	0	0
Total	16	100

One participant who did not answer this question was excluded from the counts.

### 3.3 Qualitative data: imagine a pregnancy

When asked to imagine that they or their partner were pregnant now, most participants discussed either continuing the pregnancy and parenting or having an abortion as their preferred pregnancy option. Seventeen participants clearly conveyed their desire to continue the pregnancy and parent. One male participant responded, “Oh, we would keep the baby” (20-year-old, male, first-generation immigrant). Participants preferring to continue the pregnancy and parent reported various levels of desire for a pregnancy now or in the next few months on the quantitative assessment (really or mostly do not want pregnancy, *N* = 9; sometimes want and sometimes do not want pregnancy, *N* = 3; really or mostly want pregnancy, *N* = 3; don't know or no answer, *N* = 2). Fourteen participants expressed their preference for abortion if they or their partner were pregnant now. Participants who would choose abortion reported ambivalence or strong desires against a pregnancy now or in the next few months (really do not want pregnancy, *N* = 14; sometimes want and sometimes do not want pregnancy, *N* = 2). Participants discussed how this hypothetical decision does or does not align with their personal views on abortion. One female participant expressed concordance with her views on abortion, “one of the first things would be like abortion. Because I feel like I am very—like I am for like abortion rights, and I just feel like it wouldn't be fair to bringing a child into a world where I'm not able to provide them all the things they need” (19-year-old, second-generation immigrant). Another female participant expressed discordance, “I would have to consider abortion, even though I don't feel too strongly for it, but I think in this case, it would be necessary. Like it would be something I would have to take into consideration a lot because I don't think I'd be able to take care of a child” (21-year-old, female, second-generation immigrant).

Few participants discussed adoption, and none reported adoption as their first-choice pregnancy option. One female participant discussed adoption as a second choice if she couldn't have an abortion: “I don't think I would keep it. If it was too late, I would keep it up for adoption but if it was early enough, I would have an abortion” (20-year-old, female, second-generation immigrant). When discussing adoption, two participants noted wanting to be involved in their child's life and not wanting to have someone else to raise their child: “I don't think adoption. I don't think giving up the baby would be an option. I think if I had a child, I would really want to just raise it and be involved with its life” (19-year-old, nonbinary, second-generation immigrant).

### 3.4 Qualitative data: influences on pregnancy decisions

**Figure 1:**
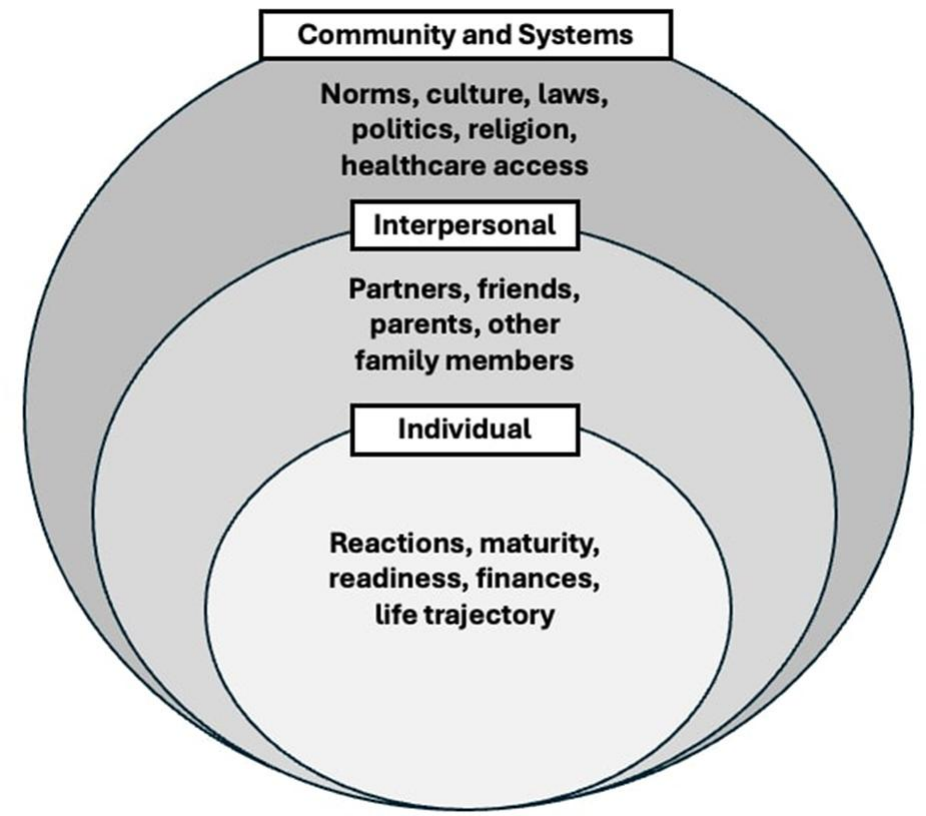
Adapted socioecological model demonstrating influences related to hypothetical pregnancy decisions that emerged from *A Crecer study* emerging adulthood interviews.

When discussing hypothetical pregnancies, participants discussed influences across socioecological levels, including individual, interpersonal, and community and systems (Figure 1). Sub-themes within each socioecological level are discussed in detail below and summarized with exemplar quotes in Table 3.

**Table 3:** When asked to imagine if they/their partner were pregnant now, participants discussed factors influencing hypothetical pregnancy decisions across multiple socioecological levels.

Individual: reactions, maturity, readiness, finances, life trajectory
***Participants described a range of reactions when asked to imagine if they or their partner were pregnant.*** • “Well first, I feel like I would be happy because I love kids. But I would also be scared because, like I said, I work, and I go to school and I wouldn't have nobody to take care of my baby.” ⚬ 19-year-old, female, first-generation immigrant ***Participants discussed the influences of personal factors (e.g., maturity, readiness, and finances) on imagined pregnancy decisions***. • “I don't think I'm ready, mentally, physically, in any aspect. I don't think I'm prepared to bear a child right now.” ⚬ 19-year-old, female, first-generation immigrant • “I just don't want to be a dad yet. I don't have the resources or the money to take care of [a baby].” ⚬ 20-year-old, male, second-generation Mexican immigrant ***Participants considered how a pregnancy in emerging adulthood would impact their life trajectory.*** • “If you have a kid now, it would probably change your entire life trajectory basically.” ⚬ 20-year-old, male, second-generation immigrant
Interpersonal: partners, friends, parents, other family
***Participants discussed the role of partners and friends in making pregnancy decisions.*** • “Personally, I would like go for it [become a parent], but ultimately, like that's what I think. And I'm kind of like of the belief of like your body, your choice. Yeah, like this is where I stand, but like I'll support whatever you want to do. That's how I think about it.” ⚬ 21-year-old, male, second-generation immigrant ***Participants described support (or lack of support) from family members, particularly parents, as a key influencer.*** • “We would need the help of our family a lot more than we do now…My parents and her parents as well. And her family mostly, because they have a lot of kids in the family right now.” ⚬ 21-year-old, male, second-generation immigrant
Community and systems: norms, culture, laws, politics, religion, healthcare
***Participants discussed cultural and community norms related to the timing of pregnancy.*** • “Like I said, for a lot of us Hispanics, teen pregnancy that's something that scared me a lot just because my parents would always say to me, we would always have this conversation. If like once you're pregnant, you're out of here. Once you are pregnant, you lose everything.” ⚬ 19-year-old, heterosexual, second-generation immigrant ***Few participants discussed other factors such as religion, laws, politics, and healthcare access as influencing hypothetical pregnancy decisions***. • “Well thankfully we live in California, it [abortion] is possible. I feel like if I lived anywhere else it'd probably be more political than anything … I'm just thankful for all the resources we have.” ⚬ 19-year-old, male, second-generation immigrant • “…but like, that's where religion comes a big play with me. Pretty much if God gave us a kid this early, then it was for a reason. So that's really what I'd like to use religion.” ⚬ 21-year-old, male, second-generation immigrant

#### 3.4.1 Individual level

*Participants described a range of reactions when asked to imagine if they or their partner were pregnant (*Table 3*).* Few participants, all of whom indicated they would continue the pregnancy and parent, expressed positive emotions, including feeling happy and joy about having a child. One participant felt neutrally: “I feel like, like I said, there's no right time for it. I feel like, if it were to happen, it's for a reason pretty much” (19-year-old, female, second-generation immigrant). Many participants reported negative emotions. Female participants shared they would feel “scared”, “terrified”, and “panicked.” These sentiments were shared among female participants who indicated they would have an abortion, those who would continue the pregnancy and those who were unsure about their hypothetical pregnancy decision. Male participants, including those who would want their partners to have an abortion, who would want their partners to continue the pregnancy, and who were unsure, said they would be “disappointed”, “scared”, “anxious”, and “stressed”.

*Participants discussed the influences of personal factors (maturity, readiness, and finances) on imagined pregnancy decisions**.*** Three participants discussed being “too young” or “not mature enough” to have a child (Table 3). Female participants, most of whom would have an abortion if they were pregnant now, expressed concerns about being “ready” to become a parent and to take care of a child (Table 3), though this sentiment was not expressed among male participants. Of these female participants, some specifically discussed the importance of stability in their mental or emotional health prior to having a baby/becoming pregnant. A female participant shared how her experience of sexual abuse influenced her perception of a pregnancy: “I don't know …  I'm like oh, a baby. But I think also being mentally fine … I'm going through all of this process of finding out that I was [sexually abused] … so I feel like being mentally fine, to be mentally okay with yourself and mentally okay to handle somebody else [a baby]” (19-year-old, female, second-generation immigrant).

Finances was a dominant theme when discussing a possible pregnancy now. Only one participant, who would continue the pregnancy and parent, reported feeling financially ready to have a child: “I'll have a full-time job, so I think I should be OK. Financially, I think I'll be OK” (19-year-old, female, second-generation immigrant). Both male and female participants discussed not having the financial resources to be the parents they want to be right now, and many participants were concerned about their current financial stability (Table 3). Participants who expressed they would decide to parent now, anticipated needing to work harder to provide for a child. A male participant who would want his partner to continue the pregnancy details how he would “hustle” to provide financially for a child: “if someone told me right now like, hey, we're going to have a kid, like the first thing I'd do is I'd go buy a lawnmower. And I'll start mowing lawns on the weekends, on the weekdays, like keep my job and try to bring in more money. I'd just hustle” (20-year-old, second-generation immigrant). A female participant who would continue the pregnancy and become a parent describes that working harder would be expected of both her and her partner: “I would definitely be working harder to make sure that I can provide for that kid. If I can't, well, he's going to find another job … if we do get an accidental pregnancy, I'd be like, okay, well, you need to find a way to f****ing pay for it, you did this” (20-year-old, female, third-generation immigrant).

*Participants considered how a pregnancy in emerging adulthood would impact their life trajectory.* Participants commented on how a pregnancy now would negatively impact their life trajectory, including future goals, education, and financial security (Table 3). One female participant shared that a pregnancy now wouldn't significantly impact her life trajectory: “I think I'd be really happy only because [by the time I'd give birth] I'm already going to have my bachelors [degree] … .That's the most important thing, for me to be done with school” (19-year-old, female, second-generation immigrant).

#### 3.4.2 Interpersonal level

*Participants discussed the role of partners and friends in making pregnancy decisions.* Male participants mentioned how they would feel about the pregnancy and their preferred outcome, but also noted it was ultimately their partner's decision (Table 3). Female participants, all of whom indicated they would have an abortion if they were pregnant now, viewed their partner being aligned with their decision as important. One of these participants specifically discussed how a disagreement about abortion affected her perception of her partner: “And he mentioned that if I were to get pregnant, then we should keep it and just try our best. And I was like, what? Like I didn't agree with that, so when he told me that, that really changed how I viewed him” (21-year-old, female, second-generation immigrant). Some participants discussed friends as important supports. Participants discussed friends having “similar values” and offering “emotional support” and “encouragement” specifically tied to pregnancy decisions.

Many participants discussed experiences of people they know when considering parenting in late adolescence and early adulthood. A female participant who would continue the hypothetical pregnancy said, “I don't know, the community I grew up in, most of them are single moms, that also encourages me that if anything happens, I feel like, I've grown up with a lot of, singular, independent women, that I would be able to do it as well” (19-year-old, second-generation immigrant). Another female participant mentioned they would receive support to continue the pregnancy from a friend who was a parent, “I would say my childhood best friend just because she's a mom. So, I know she dealt with teen pregnancy so I know she would be the one to best help me and get me through that” (19-year-old, second-generation immigrant). Few participants shared stories of friends, family members, or community members having an abortion or indicated they would seek advice from someone they knew who had an abortion. A few male participants who would want their partner to have an abortion discussed the impact of seeing people they know parent: “Because my cousin had a teen pregnancy  … Well, I know it's pretty stressful. But seeing her, I know it's pretty bad. And also she was so stressed, so depressed” (19-year-old, male, second-generation immigrant).

*Participants described support (or lack of support) from family members, particularly parents, as a key influencer.* Many participants discussed their family as an important support system if they or their partner were pregnant now (Table 3). Participants mostly discussed their parents but also mentioned siblings, cousins, aunts, and uncles. One nonbinary participant shared that they would feel comfortable going to their mom and their sister based on previous similar experiences: “I would most likely turn to my mother if anything [because] I've seen the way that she's acted with my sister and stuff, because my sister has kids … Or if anything, I could go to my sister. But I'd probably just ask my mother for help” (19-year-old, nonbinary, second-generation immigrant). A female participant who would have an abortion was thankful that she would be able to go to her mom for help in this situation: “You know like there's always—my mom, thankfully, is like a rock for me, so whenever I come to her about any issues I have, she is always making sure she's always there to reassure me first about my feelings and things like that” (19-year-old, female, second-generation immigrant). In contrast, a few female participants, all of whom would have an abortion, stated that they would not be able to turn to their family for support: “I am the type of person that I like to go through things by myself because I feel like, first of all, my parents would definitely judge or say something, they're very old school. So, I'm like no. My cousin, she would end up telling my mom somehow. So no, I would keep it to myself. I wouldn't turn to anybody” (21-year-old, female, second-generation immigrant).

Participants cited a range of likely reactions from parents (or partner's parents) about the hypothetical pregnancy. Some participants, all of whom would decide to parent, thought their family would be happy. Other participants thought their parents would be disappointed. Participants also shared that, despite this initial disappointment, their parents would be supportive: “I know that they [parents] would support me, but I understand that they would also be kind of disappointed in a way just because they understand that maybe it's too early and stuff like that” (21-year-old, male, second-generation immigrant).

Participants discussed family views on abortion, which included negative and positive sentiments. A female participant who would have an abortion explained, “I really do think they would never talk to me again. [Laughs]. Just because they all got pregnant and immediately said ‘okay well, it's time to be a mom” and I'm just kind of saying, I don't want it… My mom, my aunts, and uncles  … They're very much anti-abortion so I feel like they would feel very betrayed in that sense” (20-year-old, female, second-generation immigrant). A female participant who would have an abortion felt her family would be supportive of this decision, “I think I would turn to them [family] for support, because all of us are so vulnerable with each other in everything we've experienced. I don't think any of us have a place to judge one another if something like that happens. I know my sister has gone through something like that. I don't think there'd be any room for judgment” (19-year-old, female, second-generation immigrant).

#### 3.4.3 Community and systems level

*Participants discussed cultural and community norms related to the timing of pregnancy, including consequences of becoming pregnant “too soon” and trying to become pregnant “too late.”* Many participants were warned about the negative impacts of teen pregnancy, including one participant sharing how the experiences of her family reflect wider community norms (Table 3). A male participant emphasized the messages he was taught about pregnancy: “Like obviously don't get a girl pregnant, like that's the number one thing never to do that they always told us” (20-year-old, male, second-generation immigrant). In contrast, other participants specifically highlighted “Mexican culture” and discussed being cautioned about waiting too long for marriage and pregnancy: “And the other big thing is a lot of us, when I say us, I mean Mexican culture and stuff like that, I saw my cousins, my older cousins and my parents start having their families in their 20s, so I think that's a very big thing for me. When I'm like oh it looks like you're 30 or oh, you are too late for a family. But it's not true. I'm not saying it's true, but I feel like in my head that's something that sticks with me” (19-year-old, female, second-generation immigrant).

*Few participants discussed other factors such as religion, laws, politics, and healthcare access as influencing hypothetical pregnancy decisions.* Few participants discussed the impact of laws and politics on their imagined pregnancy decision, including the context of accessing abortion in California (Table 3). Male participants who would want their partner to continue the pregnancy and become a parent, shared the influence of religion on their decisions (Table 3). In contrast, another male participant who would want his partner to have an abortion shared how his view of abortion conflicts with that of his religious upbringing: “I think that women should have the right to abort, but on the other side, my religion doesn't think that, so I wouldn't want to be stuck with a child that I didn't plan to have” (19-year-old, male, second-generation immigrant). A few participants directly discussed access to healthcare when considering a hypothetical pregnancy, including challenges accessing health insurance and finding a clinic to access the needed healthcare.

## 4 Discussion

Our study explored the pregnancy perspectives of 41 emerging adults, almost all of whom identified as Latine, from an agricultural community in California. Participants reported a range of reactions to a hypothetical pregnancy and discussed continuing the pregnancy and parenting or abortion as preferred pregnancy outcomes. Consistent with prior research, some participants in our sample had discordant reports of pregnancy desire, perception of the pregnancy (e.g., anticipated emotions), and preferred pregnancy decision (10, 16). These findings enhance our understanding of Latine AYA's perception of pregnancy, including the complex interplay of desires, emotions, and intentions related to pregnancy. Participants discussed multiple factors across the socioecological model that influenced perceptions of a pregnancy and related decisions, emphasizing that multiple factors beyond desire to have or prevent a pregnancy shape whether a pregnancy may be acceptable. Taken together these findings enhance our understanding of how Latine emerging adults consider pregnancy and pregnancy decisions inclusive of those who experience structural barriers to accessing care, such as those who live in agricultural communities, who identify as nonbinary, and use public insurance or are uninsured.

The influence of financial circumstances on pregnancy perceptions and acceptability has been noted in qualitative work investigating these topics among urban young adult couples (3, 10), adult women who expressed not wanting more children (ever or in the next 4 years) (14), and young adult Latine women in South Florida who experienced a recent pregnancy (6). Additionally, qualitative research following the Supreme Court Decision in *Dobbs v. Jackson Women's Health Organization* found that life circumstances, including impact on their future and finances, are main factors AYAs in the United States take into account when considering abortion (20). Similarly, in a qualitative study with young adult heterosexual couples, Gomez et al. found that social advantage influenced pregnancy planning and contraceptive use (3). Participants with high social advantage discussed a desire to prevent pregnancy and used highly effective contraception due to the perception that “things will be different later” and they would eventually have the resources needed to parent (3). Conversely, socially disadvantaged participants did not find pregnancy prevention salient and used condoms or no contraception, as they doubted ever having the necessary resources to parent (3). Consistent with the literature, participants in our study considered their financial circumstances and the impact of the pregnancy on their life trajectory when discussing hypothetical pregnancies. Some participants recognized they were not in the financial situation to “plan” for a pregnancy but would “hustle” or “work harder” to provide financial stability for a child. Based on prior qualitative literature, this “hustle” narrative may be related to participants' perceived future economic/education opportunities (3) or the perception a pregnancy is a “blessing” even if raising a child would pose a substantial burden (14). This finding could also be related to familial, community, and cultural norms that shape what pregnancy decisions are normative and if abortion is considered an acceptable decision, as discussed in more detail below. Future research should continue to explore this emerging theme, focusing on how family, community, culture, socioeconomic status, and educational opportunities may influence Latine AYAs' consideration of finances in pregnancy decisions. These findings add novel insights on this topic, as we report on the perspectives of Latine young adults in an agricultural-based community in California who represent diverse relationship experiences. Furthermore, while our analysis lists finances as an “individual level” factor, it is important to recognize that financial status is inextricably linked to structural factors, including economic policy, housing policy, access to education, and racism. As such, our findings add to the literature emphasizing the influence of structural factors on pregnancy timing, pregnancy acceptability, pregnancy decisions, and reproductive self-determination (3, 4, 6, 10, 14, 20–23). This highlights the need for reproductive justice informed policy interventions aimed at ensuring young people have the financial and material resources they need to parent, when desired, and to raise their children in safe and sustainable communities (24). Such systems- and policy-interventions include universal childcare, affordable housing, financial support for educational opportunities, and employment with living wages.

Research has demonstrated that support from interpersonal relationships, such as partners and family, are important factors influencing AYA pregnancy perceptions, pregnancy acceptability, and pregnancy decisions (6, 20). Our participants discussed support from family, particularly parents, as a key influencer when considering a hypothetical pregnancy. Most of our sample reported currently living with parent(s) and/or sibling(s), which likely influenced the importance of family support in our sample. The importance of family in considering pregnancy decisions is consistent with prior work with this cohort as adolescents, which demonstrated participants preferred to talk to family about romantic relationships (“they understand me, I understand them”) (25). Consistent with findings from similar qualitative work with Latine young adult women in South Florida who had a recent pregnancy (6), many participants in our study shared concerns about their family's reactions to the hypothetical pregnancy and/or decision. However, most felt like they could go to their family for support, even if they thought their family would be initially disappointed. While peers were discussed as supports, this occurred less than discussions about family. The study's Young Adult Advisory Board provided additional context for this finding, describing they would be worried about judgement from peers or their friendships ending, particularly if considering abortion. Although this difference requires additional exploration, our findings suggest there is likely a difference in this population's perceived stability of peer vs. family relationships when navigating divergent values related to pregnancy decisions. Additionally, partners may have been discussed less often due to our participant sampling methodology, as not all of our participants had current or recent sexual partners or relationships. Given the importance of family in pregnancy perceptions and decisions, further research should explore the ways in which Latine young adults would prefer to engage parents in pregnancy options counseling with healthcare providers to inform clinical practice. This may include bringing parents into pregnancy options counseling, when desired, or supporting patients in navigating the complex family dynamics that influence pregnancy acceptability and pregnancy decisions. Healthcare providers would likely benefit from additional training in ensuring the young adult patient's autonomy is prioritized when making their pregnancy decision, especially when the patient's desires conflict with those of the parent.

Community and cultural norms can influence pregnancy acceptability (1) and pregnancy decisions (26). Experts hypothesize that norms could constrain an individual's reproductive autonomy when a norm violation results in stigma and other negative consequences (26). Furthermore, norms about sex, timing of pregnancy, and pregnancy options are pervasive in United States society and can vary based on community factors, most notably socioeconomic status and degree of religiosity (27). However, little research has described how these norms, and potential internalized stigma of teen or “unplanned” pregnancy, influence Latine AYAs' pregnancy perception and decisions. Our participants shared experiences of being warned about teen or unplanned pregnancy from a young age and named the influence of “Mexican” or “Hispanic” culture on considering timing of a pregnancy. This is similar to results from a qualitative study of Latine young adults in South Florida where participants shared feelings of personal blame related to the term “unintended pregnancy” (6). In contrast, one participant expressed concern about waiting too long to become pregnant. This adds to the findings of prior qualitative work where this concern was also expressed by urban Latine AYAs in California, particularly among participants with future uncertainty related to lack of employment opportunities and gang-related violence (23). Additionally, participants shared many stories of young adult parents from their communities, but few discussed knowing people who had an abortion. It is unclear whether the lack of examples of abortion in the community is due to a low prevalence of abortion in this community, stigma influencing acceptability of abortion as a pregnancy decision, challenges accessing abortion, or a result of abortions occurring in secret due to internalized abortion stigma (27, 28). More research to understand how lack of abortion visibility in a community can impact an individual's perception of their pregnancy decisions (e.g., what is available, what is possible, and what is consistent with community norms) could deepen interpretation of this finding. Our team will explore this theme further in the next round of qualitative interviews with the cohort. Lastly, the influence of religion on pregnancy acceptability in Latine populations has been established in quantitative research (29), and qualitative studies have explored the concept of “God's plan” or “fatalism” in this population (6, 10, 14). However, religion was only discussed by a few participants in our sample. Most of our participants were first- or second-generation immigrants, which may have influenced the importance of religion in this context, particularly among female participants, as prior research found religiosity had the most significant associations with pregnancy acceptability among third-generation Latine cisgender women (ages 18–34 years old) (29). Future research should continue to explore the norms that influence Latine AYA's reproductive goals, behaviors, and outcomes, the implications of violating these norms, and how these norms may act as coercive factors constraining available choices to further understand the context in which this population is navigating their reproductive lives.

### 4.1 Strengths and limitations

Our study has several limitations worth noting. Our small sample size and inclusion of participants from one agricultural community in California limits generalizability, especially to young adults identifying as other races or ethnicities or living in urban communities. Although we had three participants who did not identify as Latine, we are unable make comparisons between Latine young adult participants and those with other racial and/or ethnic identities. Additionally, our sample is from California, which has protective abortion policies (30). This abortion legal landscape likely influenced participants discussions of policy influences on pregnancy perceptions and decisions, which limits generalizability to young adults in states with abortion restrictions or reduced abortion access. However, it is possible that the insights from our study could be transferable to other Latine young adults living in states with similar abortion laws and in communities with established immigrant-based populations. While the community-engaged, longitudinal nature of this study and the use of similarly aged interviewers from the community likely built trust, reduced the power differential between the interviewer and the participant, and fostered an open dialogue, it is possible that social desirability bias influenced the participants' responses. The prompt may capture more immediate reactions and emotions to a hypothetical pregnancy and be insufficient fully explore the multidimensional concepts of pregnancy perspectives/acceptability. Additionally, given that the prompt explored a hypothetical pregnancy scenario and most of our participants had never been pregnant or ever had a pregnant partner, it is possible that the perspectives shared may not reflect the true perspectives or decisions of a real pregnancy or capture the real-time emotions and pressures of an actual pregnancy decision. However, the prospective nature of study adds valuable information to the existing literature given that recall bias, social desirability bias, and outcomes and social implications of an actual pregnancy could influence retrospective discussions.

### 4.2 Conclusions

Our study provides important information about the pregnancy perceptions and decisions among a sample of rural Latine young adults participating in an ongoing prospective cohort study. These findings deepen our understanding of the socioecological influences on pregnancy decision making among this population, particularly financial circumstances, perceived impact on life trajectory, family, and cultural norms. The insights on perception of abortion as a pregnancy option may be particularly valuable in an evolving abortion legal landscape in the US. Future research should continue to explore holistic frameworks to describe individuals' perspectives on pregnancy, as well as work to determine interventions that address the different socioecological levels to help individuals realize their reproductive goals.

## Data Availability

The datasets presented in this article are not readily available because the data generated for this study are qualitative interviews with potentially participant identifying information. Requests to access the datasets should be directed to aminnis@rti.org.

## Author's note

This work was presented as a poster presentation at the 2024 Society of Family Planning conference (Detroit, MI, USA; October 2024).
